# Regime Shift in Fertilizer Commodities Indicates More Turbulence Ahead for Food Security

**DOI:** 10.1371/journal.pone.0093998

**Published:** 2014-05-01

**Authors:** James J. Elser, Timothy J. Elser, Stephen R. Carpenter, William A. Brock

**Affiliations:** 1 School of Life Sciences, Arizona State University, Tempe, Arizona, United States of America; 2 Flyr, Inc., San Francisco, California, United States of America; 3 Center for Limnology, University of Wisconsin, Madison, Wisconsin, United States of America; 4 Department of Economics, University of Wisconsin, Madison, Wisconsin, United States of America; 5 Department of Economics, University of Missouri, Columbia, Missouri, United States of America; Centro de Investigación y de Estudios Avanzados del IPN, Mexico

## Abstract

Recent human population increase has been enabled by a massive expansion of global agricultural production. A key component of this “Green Revolution” has been application of inorganic fertilizers to produce and maintain high crop yields. However, the long-term sustainability of these practices is unclear given the eutrophying effects of fertilizer runoff as well as the reliance of fertilizer production on finite non-renewable resources such as mined phosphate- and potassium-bearing rocks. Indeed, recent volatility in food and agricultural commodity prices, especially phosphate fertilizer, has raised concerns about emerging constraints on fertilizer production with consequences for its affordability in the developing world. We examined 30 years of monthly prices of fertilizer commodities (phosphate rock, urea, and potassium) for comparison with three food commodities (maize, wheat, and rice) and three non-agricultural commodities (gold, nickel, and petroleum). Here we show that all commodity prices, except gold, had significant change points between 2007–2009, but the fertilizer commodities, and especially phosphate rock, showed multiple symptoms of nonlinear critical transitions. In contrast to fertilizers and to rice, maize and wheat prices did not show significant signs of nonlinear dynamics. From these results we infer a recent emergence of a scarcity price in global fertilizer markets, a result signaling a new high price regime for these essential agricultural inputs. Such a regime will challenge on-going efforts to establish global food security but may also prompt fertilizer use practices and nutrient recovery strategies that reduce eutrophication.

## Introduction

The human population has more than doubled during the past fifty years, during which time *per capita* food availability has nevertheless increased [1]. This was made possible by the “Green Revolution”, the large-scale expansion and intensification of agricultural activity that included extension of cultivated lands, development of high yield crop varieties, increased irrigation, and heavy application of inorganic fertilizers to supply nitrogen (N), phosphorus (P), and potassium (K). However, many concerns about the present and future sustainability of these activities have been raised [2]; in this paper we focus on dimensions related to inorganic fertilizers [3]. Large amounts of N and P fertilizers are used in agriculture each year, with much of the added N and P being lost from farms and livestock operations and leading to widespread degradation of the quality of fresh waters as well as damage to coastal marine ecosystems [4]. Furthermore, concerns about the continued availability and affordability of inorganic fertilizers, especially those based on P, have recently been raised following the several-fold increase in the price of phosphate rock and fertilizers in 2007 and 2008 [5]. Such price increases are of particular importance because small conventional stakeholder farmers in developing countries often lack the financial resources to buffer such increases in needed input commodities, such as fertilizers. As these farmers are a significant proportion of the global undernourished population, increases in inorganic fertilizer prices can diminish important components of food security [5]. Reserve estimates for phosphate rock have recently been revised substantially upward [6]. Furthermore, there is always potential for adaptive responses of technology (such as nutrient recovery or crop biotechnology approaches) and market systems to emerging geological scarcity [7]–[9]. Nevertheless, converging trends suggest that the fertilizer/food system will come under increased pressure in coming decades. First, to assure the food security of the global population in 2050, food production may need to double [10]. Second, growing worldwide affluence means that global diets will include increasing amounts of meat [11]; meat-intensive diets require disproportionately more nutrients (N, P) to produce [12]. Third, non-food uses of nutrient elements (e.g. N and P to produce biofuels [13]; P to produce lithium-phosphate batteries) are increasing dramatically and are beginning to impose novel market pressures on the fertilizer sector. While various approaches for diversifying the sources of P for fertilizer production are under investigation (e.g. P recycling from human waste and other waste streams; [14]), the time-scales of development and adoption of these strategies are not yet clear and thus concern remains about scenarios for future global P dynamics [15].

Because of the essential nature of fertilizers in supporting the food system, it is important to gain insight into overall trends and patterns of variability in fertilizer prices. This is an especially challenging task given how deeply N and P are embedded within an increasingly complex and globalized socioeconomic ecosystem. One way to achieve such insights is to examine time series for significant breakpoints in temporal trends as well as for symptoms of possible “critical transitions” that accompany, and sometimes presage, regime shifts in diverse domains from ecology (e.g.: fishery collapse) to medicine (e.g.: heart attack) [16], [17]. Regime shifts may be brought about by external perturbations but many of them can be explained by critical transitions in system dynamics [18], [19]. In economic analyses, basic theory of asset markets (called the Efficient Market Hypothesis, EMH) argues that strong market forces make such price changes roughly unpredictable over time [20]. EMH theory does not rule out rapid up bursts or rapid declines nor does it limit volatility bursts or persistence in volatility; indeed, *a posteriori* analyses may detect significant change points. Here we focus on such change points and statistical symptoms of critical transitions near change points and provide the first such application of these approaches to time series related to agricultural input commodities (fertilizers) and crop commodities.

Our primary research goals were two-fold: first, to identify possible breakpoints in the data series for agricultural fertilizers that mark significant changes in market conditions and, second, to assess the possibility that these commodities are affected by nonlinear system dynamics and thus undergoing critical transitions. To do this we apply time series analytical methods [21] to phosphate rock price data along with data for two other fertilizer commodities, potassium (K) and urea. To assess if changes in fertilizer dynamics might be driven by associated regime shifts in key crops, we also used similar methods to evaluate the price time series for three major crop commodities: maize, rice, and wheat. To see if fertilizer dynamics might be associated with dynamics of energy prices we analyzed the time series for petroleum. We also considered the dynamics of nickel, as an indicator of non-agricultural volatility patterns, and of gold, as an indicator of overall financial volatility in the global system. Other investigators have previously considered volatility patterns in commodity prices with special attention on cross-sector price transmission [22]–[24]. However, our analysis focuses less on issues of transmission and elasticity (e.g. how do changes in fertilizer prices manifest in changes in food prices, or vice versa?) but more on issues related to characterizing the qualitative nature of underlying dynamics in the systems that underpin key commodities (e.g. linear vs nonlinear dynamics) and to identifying potential breakpoints in those dynamics, as these may signal regime shifts in those commodities. Our approach is mainly descriptive and assessments of all of the complex mechanistic inter-relations among these series await future analyses.

## Methods

We analyzed several decades of monthly price time series for (i) three primary components of industrial fertilizer, phosphate rock, potassium (K), and urea (an organic molecule rich in N); (ii) three food commodities, maize, rice, and wheat; and (iii) three non-agricultural commodities, petroleum, gold, and nickel. The non-agricultural commodities were chosen in order to evaluate if possible changes identified in the agricultural realm were simply mirroring those ongoing in the energy (petroleum), financial (gold), or general industrial (nickel) sectors. Our analysis focuses on raw input commodities (e.g. phosphate rock) instead of finished products (e.g. tri-, di-, sodium, potassium phosphate fertilizers) because we wished to more easily make comparisons among input commodities in a way that was less likely to be influenced by indirect effects that are felt for finished products (e.g. costs of transport, marketing, rents, overhead, etc). For each time series, we fit an autoregressive change point model to the series, filtered residuals using a general autoregressive conditional heteroskedasticity (GARCH) model, and subjected GARCH residuals to a test for linearity [27]. We also computed rolling-window variance and autocorrelation, indices that are commonly used as indicators of regime shifts [21], [28]. Further details are provided in online Supplemental Information.

Monthly data for each commodity were obtained from the World Bank (data.worldbank.org/data-catalog/commodity-price-data). All series were adjusted for inflation using the US consumer price index time series for all urban consumers (downloaded 21 May 2013). Based on normal probability plots, time series were log_10_-transformed prior to analysis. All time series were subjected to 36-month rolling window calculations of variance and autocorrelation [21], [27], [28]. Autocorrelation statistics were transformed to autocorrelation time, −1/ln(AC) [29].

Critical transitions imply the operation of nonlinear or non-stationary mechanisms in the stochastic dynamical system, i.e. stochastic process, that underpins a time series [27]. In this paper, we define stochastic processes as “nonlinear-in-mean” if the underlying system is nonlinear in past values of “x” when external disturbances are set to zero. In addition, the stochastic process is “nonlinear-in-time trend” if the underlying dynamical system is a nonlinear function of time. To detect such systems, one can fit models that are linear but also have additional nonlinear terms as functions of past “x” and of time and evaluate the statistical significance of the models’ nonlinear terms; the residuals of such models are also explored for further structure [27], [30]. We followed this strategy here, fitting GARCH models [20] to the residuals of our model fits, and testing the standardized residuals for “extra structure” missed by the fitted GARCH models [27], including regime switches in variance.

Plots of autocorrelation and partial autocorrelation functions (PACF) indicated that all series required autoregression (AR) correction. Change point models with autoregressive terms were fit to first-differenced data by maximum likelihood. The best-fitting models were selected using Akaike’s information criterion [31] as well as diagnostics for residual time series. Models with residuals exhibiting no predictable structure, especially non-significant PACFs, were selected for further analysis. Residuals of these models were fitted to a GARCH model. GARCH residuals were renormalized and analyzed by the bootstrap BDS (Brock-Dechert-Scheinkman) test using 1000 iterations. The BDS tests the null hypothesis that the renormalized GARCH residuals are identically distributed and independent [32]; rejection of this null hypothesis implies that some structure remains in the series, including potential nonlinearities.

## Results

**Figure 1:**
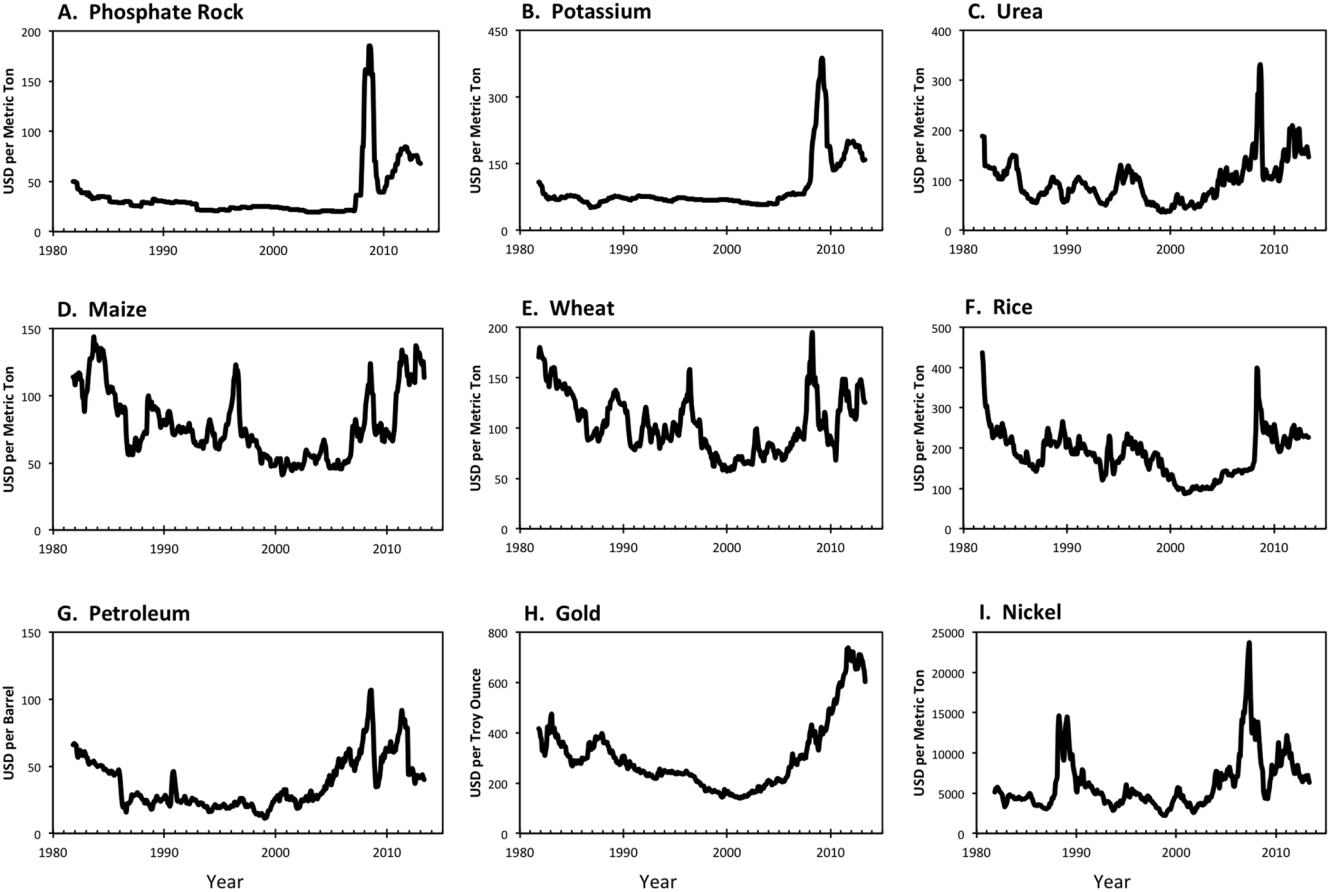
Commodity price time series from 1981 to 2011, corrected for inflation to 1982 price.

**Figure 2:**
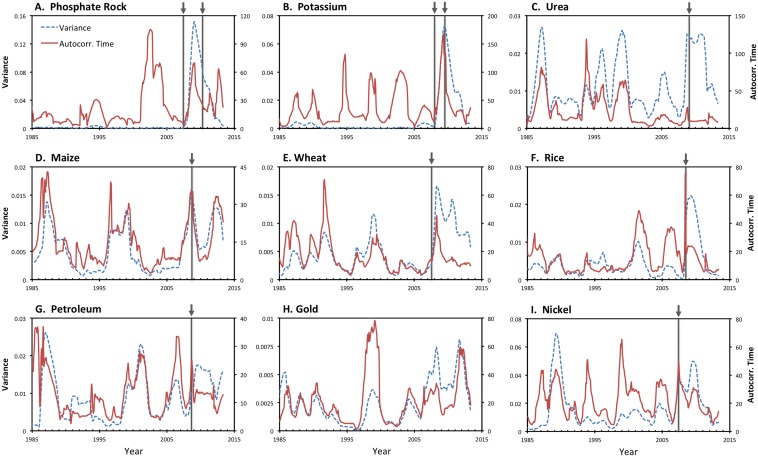
Temporal breakpoints and statistical indicators of critical transition for nine commodities. Arrows and vertical lines show statistically significant change points. Variance (blue, dashed) and autocorrelation time (red, solid) for log10-transformed data were computed for 36-month rolling windows. Autocorrelation time is the negative inverse of the natural logarithm of the autocorrelation coefficient [42].

All nine of the commodities studied exhibited substantial dynamics (Figure 1). Indeed, we detected significant change points for each commodity except for gold (Table 1, Figure 1). All of the significant change points were associated with peaks in autocorrelation or variance (Figure 2). However, it is difficult to evaluate the statistical significance of the autocorrelation and variance time series. We used the BDS test (Table 1) for significance tests on the null hypothesis that each price time series followed linear processes. Significant departures from linear behavior were detected for all three fertilizer components, as well as for rice (Table 1 and Table S1 and Table S2 in File S1).

**Table 1:** Change points (if any) in years and results of the BDS test for the commodity time series.

		BDS *P* values	
Commodity	Change Points	(3 values of epsilon)	Inference
Phosphate Rock	May 2007, March 2010	0, 0, 0	Change points, not linear
Potassium	January 2008, July 2009	0.015, 0.008, 0.014	Change points, not linear
Urea	January 2009	0.02, 0, 0	Change point, not linear
Rice	March 2008	0.054, 0.011, 0.032	Change point, not linear
Maize	September 2008	0.19, 0.14, 0.15	Change point, linear
Wheat	August 2007	0.61, 0.78, 0.91	Change point, linear
Petroleum	September 2008	0.23, 0.23, 0.25	Change point, linear
Gold	None	0.92, 0.92, 0.78	No change point, linear
Nickel	April 2007	0.61, 0.26, 0.42	Change point, linear

BDS tests the null hypothesis that the standardized residuals of the change point model come from a stationary stochastically independent process. A low *P* value rejects the hypothesis of stationary independence. ‘Inference’ is our interpretation of the statistics. Change point model fits, GARCH fits, and results of bootstrapped BDS *P* values are presented in Supplementary Information.

Statistical analysis of the phosphate rock price time series (Figure 1A) identified three distinctive time intervals (Table 1): Regime 1 (before May 2007), Regime 2 (May 2007–March 2010), and Regime 3 (after March 2010). In addition, for phosphate rock, both variance and autocorrelation rose and fell together during 2007–2010 (Figure 2A) and the BDS test for phosphate rock strongly rejects the hypothesis of linearity. The shift in 2007 represents a transition from a long phase of gradual change (Regime 1) to a sharp increase followed by a steep decrease (Regime 2) while the shift in 2010 is associated with the more gradual rise and subsequent recent decline near the end of the series (Regime 3). Thus, the behavior of the phosphate rock time series reveals two major breakpoints in recent years (including the earliest breakpoint detected of the commodities we studied) and statistical behavior that is consistent with a critical transition under nonlinear dynamics. However, we note that the distinctiveness of Regime 3 is somewhat uncertain given the limited amount of data following the March 2010 breakpoint.

Similar to phosphate rock dynamics, the K price time series also exhibits two change points (January 2008 and July 2009; Figure 1B) although the timing of these events is not coincident. As for phosphate rock, the BDS test for the K time series was consistent with nonlinear behavior, although the BDS phosphate rock values were somewhat higher (Table 1). The urea price time series contained a significant shift at the beginning of 2009 (Figure 1C) that was also marked by increases in variance (Figure 2C). The BDS test suggests that the urea time series cannot be explained entirely by linear processes (Table 1).

The three crop commodities considered also had significant change points (Table 1). For wheat, a significant disruption was detected in August 2007 while those for rice and maize came later (March 2008 and September 2008, respectively). In contrast to fertilizers, indications of nonlinear dynamics were weaker in the crop commodities. Indeed, only rice showed statistically significant BDS tests but the *P* values (0.011–0.054) were higher than those seen for the fertilizer commodities (Table 1 and Table S1 and Table S2 in File S1).

The non-agricultural commodities we evaluated showed behaviors distinct from those of the agricultural commodities, and especially of urea, K, and phosphate rock. Petroleum price had a change point in September 2008 (Figure 1D). While this change point was associated with rising variance and autocorrelation time (Figure 2D), the BDS test for petroleum was not significant and thus the dynamics are consistent with linear processes. Gold fit a seasonal autoregressive model with no change points (Figure 1E, Table 1). While there were correlated changes in variance and autocorrelation after 2005 (Figure 2E), the BDS test for gold was not significant. Nickel dynamics exhibited a significant change point in 2007 (Table 1, Figure 1F). While nickel prices also exhibited coherent fluctuations in variance and autocorrelation time after 2006 (Figure 2F), the BDS result was consistent with linear processes (Table 1).

Based on these analyses, we argue that the evidence suggests that there was a temporary disruption in fertilizer markets during the 2007–2009 period that was especially notable in the phosphate rock market between March 2007 and July 2009, during which prices rose to very high levels and then dropped precipitously to levels somewhat higher than those preceding. This volatility in phosphate rock prices was followed by what appears to be a regime change to a phase of higher prices. Overall, high and increasing prices after 2009, along with signals in the variance structure, suggest the arrival of a new phosphate rock regime that is increasingly influenced by Hamilton’s “scarcity price” [25], a scenario that will challenge attempts to achieve food security in coming decades [26].

## Discussion

We assessed whether price dynamics of key agricultural input commodities {nutrient fertilizers: urea, potassium (K), and especially phosphorus (P) as phosphate rock} during recent decades contained evidence for significant change points as well as for critical transitions [17], [33]. We also determined if such dynamics were distinct from the behavior of crop commodities as well as non-agricultural commodities, such as those connected to transportation (petroleum), financial (gold), or industrial (nickel) sectors. Our most significant finding is that the price dynamics of fertilizers appear to be unique among these time series. While all of the commodities showed considerable turbulence from 2006 to the present, all three fertilizer components, and especially phosphate rock, displayed multiple change points as well as significant departures from linearity during this period. Among the other commodities, only the dynamics of rice were suggestive of nonlinear dynamics associated with a significant change point but these indications were weaker than those seen for the fertilizers.

As a finite geological resource rises in price, it is likely that technological advances in resource extraction, conservation, or recycling will occur. Thus, the prospect of ‘peak phosphorus’ is a contested idea. Two recent articles [25], [34] seek ways to detect evidence of “peaking” (e.g. “peak oil”) or impending increased scarcity in an exhaustible resource using time series analyses. The basic Hotelling theory discussed by Smith [34] predicts a general uptrend in price due to an increasing scarcity component of the price above and beyond that which is set by shorter term forces of supply and demand [25]. Hamilton notes that this scarcity component is likely to increase as the resource becomes scarcer and harder to extract, as new geographic sources diminish, and as demand rises [25]. Here we do not attempt to evaluate the plausibility of ‘peak phosphorus’. Instead, in the case of P we have focused on the price of phosphate rock in relation to the behavior of other key fertilizer components, rather than their geological abundances [35]. This focus is appropriate for the economics of industrial agriculture, and relevant in considerations of the food security of the world’s poorer peoples who cannot independently afford inorganic fertilizers even at current prices.

For example, we find statistical evidence for three regimes in phosphate rock prices (Table 1, Figures 1 and 2). The first regime of stable and slightly declining prices is quite precisely fitted because it has a relatively large number of observations and rather consistent behavior over time; it lasted until approximately March 2007. The second regime (approximately March 2007 to February 2009) was a turbulent one with a rapid rise in prices followed by an equally rapid drop in prices. The final regime from February 2009 through 2011 exhibited a more moderate rise in prices. Because the sample size is limited after 2009 we cannot be certain if this third phase of gradually rising prices will be sustained or give way to something different. The dynamics of potassium (K) prices also were consistent with nonlinearity but the change points for potassium (K) followed those for phosphorus by about half a year. Urea underwent a change point in 2009, at the end of the turbulent period for phosphorus prices, and its dynamics also indicate nonlinearity. While evidence of change points was seen in the dynamics of several of the non-agricultural commodities we considered, no clear indications of nonlinearity were documented among these (only rice prices showed some signs of nonlinear behavior).

For an exhaustible resource like phosphate rock or oil, economists have developed the concept of a “scarcity price.” This refers to the underlying price that a unit of the resource would fetch under average conditions of short run supply and demand (also sometimes called the “royalty price”, [36]). This component of the current observed market price would be zero if known resources of the exhaustible resource were infinite. Though provisional, our analyses suggest that fertilizer markets, and especially that for phosphate rock, have entered a regime in which there is a scarcity price operating [25], [34]. The future will reveal whether this scarcity regime is stable or only the prelude to yet another, as yet unknown, regime shift. These recent shifts, with their rapid bursts in price followed by rapid declines, can be viewed as a warning sign that similar large disruptions in fertilizer markets could occur in the future. In the case of phosphate rock, our analyses show that the volatility of residuals of fitted models for phosphate rock prices has increased in the last two regimes relative to the first. This is yet another piece of evidence that the underlying fundamentals of the phosphate rock market have changed, a change with significant implications for global food security.

The causes of these shifts cannot be identified via time-series analysis alone. Mechanistic inferences would instead require detailed macroeconomic modeling combined with fine-scale data about shifts in supply and demand for the various commodities. Such analyses are beyond the scope of this study, whose main goal was to characterize the volatility dynamics of these commodities and test for possible change points in the market systems connected to them. However, the differences we observed among the commodity time series are suggestive of possible contributing factors. First, the disconnect between the fertilizer commodity dynamics and those for the non-fertilizer commodities suggests that the underlying mechanisms are not tightly connected to demand-side drivers from major crops (as indexed by rice, maize, wheat), to shifts in energy costs (as indexed by petroleum), to general economic volatility (as indexed by gold), or to overall industrial activity (as indexed by an industrial material, nickel). This is somewhat surprising, given the previous documentation of price transmission between petroleum and “fertilizer” commodities [22], [23]. It important to note, however that in these previously published analyses of price transmission, “fertilizer” corresponded to finished tri-sodium phosphate and not to phosphate rock, as in our analysis. Instead, our work suggests that market and sociopolitical forces localized within the agricultural sector itself (including the fertilizer industry) appear sufficiently strong to impose their own flavor on the emerging dynamics seen in P, K, and N commodity prices. Nevertheless, cross-sector analyses using bivariate and multivariate approaches (e.g. [24], [37], [38]) to evaluate connections among fertilizer, crop, and petroleum volatilities may be informative and await further analysis. Second, differences among the agricultural commodity series themselves provide some hints about potential contributing factors. Bearing in mind differences in how these other fertilizer components are sourced (e.g. beyond inorganic fertilizer, N inputs to farm fields can also involve rotation strategies that promote N fixation by legumes), if dynamics were driven largely by demand-side forces we would expect similar temporal behaviors either when comparing crops (maize, rice, wheat) to fertilizers (N, P, K) or when comparing the fertilizers to each other (since demand for these key fertilizer components is strongly coupled by their joint essentiality in supporting high crop yield). However, crop change points, as well as those for N and K, were significantly delayed with respect to those for P. Notably, the three fertilizer components and especially P showed signs consistent with nonlinear dynamics but, among the crop commodities, only rice prices showed evidence of nonlinearity and this was relatively weak. We speculate that local and regional factors associated with the supply sectors for each fertilizer component result in disjunct dynamics because the source countries are different for each element. For example, P supplies are increasingly dominated by activities and policies in Morocco and China while K supplies are dominated by events in their main producers, Canada and Russia.

Time-series analyses are, of course, retrospective and it is problematic to forecast future trajectories from past events, as noted in particular case of the dynamical P system [7]. Nevertheless, the question naturally arises about the likelihood of a return to earlier regimes of low, relatively stable fertilizer prices. One natural market response to rising prices for an extractable resource such as phosphate rock is expansion of production and development of previously economically non-viable resources [25], a trend that is already underway for phosphate rock [7]. However, such responses beg the question if the focus is not on the trajectory of overall production (e.g. the possibility of “peak phosphorus”) but instead is on the trajectory of price, as development of these “new” reserves involves exploitation of deposits that have lower resource concentrations and/or higher contaminant levels, lie under greater overburden, or are located further from fertilizer production facilities and markets. All of these imply higher costs and suggest that a near-term return to the earlier, low-price, regimes is unlikely, especially in light of projected increases in demand over the next several decades due to increasing human population size, rising meat consumption, and non-agricultural uses of P [10], [12], [13]. In the case of phosphate rock, this expectation of sustained high prices in the near-term (next decade or two) is shared by industry analysts despite documented expansion of exploration and exploitation [39]. Morocco’s emerging oligopoly over global reserves of phosphate rock [40] may also help to sustain high P prices. However, transformational innovation in crop nutrient use efficiency and in nutrient recycling approaches in the food system [14], [41] may be one pathway for a return to the earlier situation, a transition that would improve fertilizer access for all who need it and thus enhance global food security. Such a transition would also reduce nutrient exports from agricultural systems and improve water quality.

Put simply, the evidence presented here confirms for the first time that fertilizer prices have undergone unique patterns of recent volatility and have moved into a new high price regime. That is, an era (*the* era?) of cheap inorganic fertilizer appears to be over. The persistence of this regime will depend on hard-to-forecast transitions to new technologies and strategies for improved crop nutrient use and for nutrient recycling via reclamation from human, animal, and crop waste streams [14]. Importantly, our analysis indicates that the extreme price fluctuations seen in recent years are not merely the rise and fall of prices in “normal” (i.e. linear) market interactions. Instead, they are symptoms of the operation of a nonlinear system and portend the possibility of similar instabilities in the future. The potential continuation of this regime of rising and unpredictable prices has adverse implications for farmers and consumers in both developed and developing countries. Given the close connections between food security and national security, governmental and non-governmental institutions may wish to consider measures to head off further escalation of fertilizer prices and to stabilize their dynamics. Such measures could involve implementing fertilizer stockpiles analogous to the USA’s strategic petroleum reserve (a concept first proposed by US President Franklin D. Roosevelt) or encouraging development of diversified local and regional sources of fertilizer from recycled sources (sewage, animal waste, food waste, etc.). A nutrient recycling strategy will have the added benefit of improving water quality in freshwater and marine ecosystems by reducing nutrient runoff from farms, livestock, and cities.

## Supporting Information

File S1 This file contains Tables S1 and S2. **Table S1.** Parameter estimates for change point models. **Table S2.** Analysis of residuals from change point models by GARCH and BDS.
(DOCX)
