# Supplementary material for: Regime Shift in Fertilizer Commodities Indicates More Turbulence Ahead for Food Security
Source: PLoS One. 2014 May 1;9(5):e93998. doi: 10.1371/journal.pone.0093998 (PMC4006770; doi:10.1371/journal.pone.0093998)
Supplement: File S1 — This file contains Tables S1 and S2. Table S1. Parameter estimates for change point models. Table S2. Analysis of residuals from change point models by GARCH and BDS. (DOCX) [file pone.0093998.s001.docx]

**Supporting Information**

Time series models were fitted to change point models. Models presented in Table S1 were selected on the basis of Akaike’s Information Criterion and diagnostics for residuals, including autocorrelation and partial autocorrelation functions. The goal was to obtain models that fit well and removed obvious structure from the residuals.

Phosphate rock had significant change points in years May 2007 and March 2010. These correspond, respectively, to the start of the recent interval of volatility and the start of the interval that appears to show a smooth rise and a recent modest decline. Potassium had significant change points in January 2008 and July 2009. Urea had a significant change point in January 2009, which marks the transition after the sharp drop that began in year mid-2008.

Each of the three crop commodities showed one change point (wheat in August 2007, rice in March 2008, and maize in September 2008).

Petroleum had a significant change point in September 2008, corresponding to the sharp drop in prices that began in year June 2008. The rough similarity to the change in urea prices, and the fact that petroleum and other fossil fuels are essential for converting atmospheric dinitrogen to urea and other fertilizers, makes it tempting to draw a connection between the dynamics of these two commodities.

Gold had no discernible change points. Nickel had a change point in April 2007, corresponding to a decline in prices followed by a phase of slowly rising prices.

The residuals from change point models were fitted to GARCH models to remove discernible patterns in the variance, and then subjected to BDS test (Table S2). The BDS P value is the P value for the null hypothesis that the standardized residuals of the change point model come from a stationary stochastically independent process (or more specifically that the residuals, after GARCH filtering, are identically and independently distributed). A low P value rejects the hypothesis of stationary independence and is indicative of non-linearities.

Time series for all three fertilizer commodities (potassium, urea, and especially phosphate rock) showed evidence that they are not linear. However, only one of the crop commodities (rice) showed signs of non-linear dynamics and its BDS P values (0.011 - 0.054) were somewhat high compared to those for the fertilizers. None of the non-agricultural commodities show signs of non-linearity (BDS P values >0.23).

Table S1. Parameter estimates for change point models. For each commodity, we present parameter estimates (standard error SE in parentheses) for AR terms, timing of change points (in years) as well as the mean and standard error of each change point effect, and residual SE of the change point model. All computations were performed in R version 2.15.3, using the function ‘arima’.

| **Commodity** | **AR(1)** | **AR(2)** | **AR(3)** | **AR(4)** | **AR(5)** | **AR(6)** | **Change points** | **Model s.e.** |
| --- | --- | --- | --- | --- | --- | --- | --- | --- |
| Phosphorus | 0.19 (0.05) | 0.11 (0.05) | 0.28  (0.05) | -- | -- | -- | May 2007, Mar 2010  Means 0.16, 0.23  (0.02, 0.03) | 6x10^-4^ |
| Potassium | 0.26  (0.05) | 0.17  (0.05) | 0.04  (0.05) | 0.13  (0.05) | -- | -- | Jan 2008, July 2009  Means 0.1189, -0.0429  (0.0122, 0.0176) | 1.5x10^-4^ |
| Urea | 0.36  (0.05) | -0.08  (0.05) | -- | -- | -- | -- | Jan 2009  Mean -0.009  (0.034) | 1.2x10^-3^ |
| Rice | 0.41  (0.05) | -.16  (0.05) | -- | -- | -- | -- | March 2008  Mean 0.1439  (0.02) | 4.9x10^-4^ |
| Maize | .24  (.05) | -- | -- | -- | -- | -- | Sept 2008  Mean -0.09  (.02) | 6x10^-4^ |
| Wheat | .26  (.05) | -- | -- | -- | -- | -- | August 2007  Mean 0.08  (.02) | 5.7x10^-4^ |
| Petroleum | 0.26  (0.05) | -0.08  (0.05) | 0.08  (0.05) | -0.11  (0.05) | -0.006  (0.05) | -0.106 (0.05) | Sept 2008  Mean -0.1024  (0.035) | 1.2x10^-3^ |
| Gold | 0.12  (0.05) | -0.11  (0.05) | -- | -- | -- | -- | none | 2.8x10^-4^ |
| Nickel | 0.33  (0.05) | -- | -- | -- | -- | -- | April 2007  Mean -0.073  (0.034) | 1.2x10^-3^ |

Table S2. Analysis of residuals from change point models by GARCH and BDS. After GARCH analysis but prior to BDS analysis, residuals from GARCH were renormalized as ε_R_ = log[ε_G_^2^/var(ε_G_)] where subscripts R and G denote renormalized residuals and GARCH residuals, respectively. BDS tests the hypothesis that residuals are independently and identically distributed (IID) after all linear structure has been removed. In BDS, rejection (low P value) of the IID hypothesis indicates remaining predictable patterns that are not explained by linear models. All computations were performed in R version 2.15.3 using the ‘tseries’ library functions ‘garch’ and ‘BDS’.

|  | **GARCH parameters (intercept, GARCH, ARCH)** | | | **P value from Bootstrapped BDS test**  **for 3 values of epsilon** | | |
| --- | --- | --- | --- | --- | --- | --- |
| **Commodity** | **a_0_** | **a_1_** | **b_1_** | **0.5 s_G_** | **0.75 s_G_** | **1 s_G_** |
| Phosphorus | 2.2x10^-5^  (3.3x10^-6^) | 0.14  (0.019) | 0.84  (0.02) | 0 | 0 | 0 |
| Potassium | 5.9x10^-7^  (1.7x10^-6^) | 0.51  (0.066) | 0.73  (0.02) | 0.015 | 0.008 | 0.014 |
| Urea | 3x10^-4^  (8x10^-5^) | 0.11  (0.04) | 0.57  (0.19) | 0.02 | 0 | 0 |
| Rice | 7.5x10^-5^  (2.3x10^-3^) | 0.13  (.05) | 0.71  (.08) | 0.054 | 0.011 | 0.032 |
| Maize | 5.7x10^-4^  (1x10^-3^) | 0.03  (.05) | 4.4x10^-14^  (1.75) | 0.188 | 0.142 | 0.146 |
| Wheat | 1.47x10^-4^  (4x10^-5^) | 0.3  (.07) | 0.47  (0.1) | 0.608 | 0.776 | 0.913 |
| Petroleum | 1x10^-3^  (2.6x10^-4^) | 0.15  (0.04) | 1.6x10^-14^  (0.2) | 0.23 | 0.233 | 0.249 |
| Gold | 1.93x10^-5^  (7x10^-6^) | 0.14  (0.04) | 0.8  (0.05) | 0.921 | 0.916 | 0.778 |
| Nickel | -- | -- | -- | 0.606 | 0.259 | 0.422 |
